# Matrix Metalloproteinase-1 and Acid Phosphatase in the Degradation of the Lamina Propria of Eruptive Pathway of Rat Molars

**DOI:** 10.3390/cells7110206

**Published:** 2018-11-10

**Authors:** José Paulo de Pizzol Júnior, Estela Sasso-Cerri, Paulo Sérgio Cerri

**Affiliations:** 1 Department of Morphology and Genetics, Federal University of São Paulo (UNIFESP), 04021-001 São Paulo, SP, Brazil; jpaulopizzol@gmail.com; 2 Laboratory of Histology and Embryology-Araraquara, School of Dentistry, São Paulo State University (UNESP), 1680 Centro, CEP 14801–903 Araraquara, SP, Brazil; estela.sasso@unesp.br

**Keywords:** eruptive pathway, matrix metalloproteinase-1, acid phosphatase, ultrastructure, lamina propria, birefringent collagen

## Abstract

The comprehension of dental pathogenesis and disorders derived from eruption failure requires a deep understanding of the molecular mechanisms underlying normal tooth eruption. As intense remodelling is needed during tooth eruption, we hypothesize that matrix metalloproteinase-1 (MMP-1) and acid phosphatase (ACP) play a role in the eruptive pathway degradation. We evaluated MMP-1-immunoexpression and the collagen content in the lamina propria at different eruptive phases. Immunohistochemistry and ultrastructural cytochemistry for detection of ACP were also performed. In the maxillary sections containing first molars of 9-, 11-, 13-, and 16-day-old rats, the birefringent collagen of eruptive pathway was quantified. MMP-1 and ACP-2 immunohistochemical reactions were performed and the number of MMP-1-immunolabelled cells was computed. Data were analyzed by one-way ANOVA and Tukey post-test (*p* ≤ 0.05). ACP cytochemistry was evaluated in specimens incubated in sodium β-glycerophosphate. In the eruptive pathway of 13- and 16-day-old rats, the number of MMP-1-immunolabelled cells increased concomitantly to reduction of collagen in the lamina propria. Enhanced ACP-2-immunolabelling was observed in the lamina propria of 13- and 16-day-old rats. Fibroblasts and macrophages showed lysosomes and vacuoles containing fragmented material reactive to ACP. MMP-1 degrades extracellular matrix, including collagen fibers, being responsible for the reduction in the collagen content during tooth eruption. The enhanced ACP activity at the mucosal penetration stage indicates that this enzyme plays a role in the degradation of remnant material, which is engulfed by macrophages and fibroblasts of the eruptive pathway. Therefore, enzymatic failure in the eruptive pathway may disturbs tooth eruption.

## 1. Introduction

In the orthodontic clinic, the adequate diagnosis of eruption disorders is essential for the correct management of the orthodontic problem [1,2]. The eruption failure cannot be classified based only on clinical characters, but the mechanism by which this pathological condition occurs, including possible physical and/or biological defects in the eruption process, should be considered and investigated [3]. Eruption disorders are in general difficult to diagnose given the scarce studies on the eruptive process [4]. Therefore, the better understanding of the pathogenic conditions and disorders derived from eruption failure depends on a complete understanding of the specific molecular mechanisms underlying normal eruption [3,5].

Tooth eruption is the phenomenon of movement of the tooth germ from it intraosseous position into occlusion position [6,7]. The tooth eruption is divided into five phases: pre-eruptive, intraosseous, mucosal penetration, pre-occlusal and post-occlusal eruption [6,8]. Accentuated structural changes in the eruptive pathway occur during the intraosseous and mucosal penetration stages [9,10]. The intraosseous stage begins with the axial movements of the tooth germ leading to the process of bone resorption, mainly in the occlusal portion of the bone crypt [6,8]. At the intraosseous phase, it has been demonstrated that the cells of the dental follicle release several factors and cytokines such as colony-stimulating factor-1 (CSF-1), monocyte chemotactic protein-1 (MCP-1) and receptor activator of NF kappa B ligand (RANKL) [11,12,13]. These molecules stimulate the recruitment and differentiation of mononuclear cells into osteoclasts, which promote an intense bone resorption, particularly in the occlusal portion of the bone crypt [14]. Moreover, we demonstrated a significant concomitant increase in the number of mast cells and osteoclasts in the eruptive pathway, indicating that mast cells seem to participate in the recruitment of osteoclasts and, consequently, in the bone resorption [9]. During the mucosal penetration phase, intense structural changes occur in the lamina propria of the eruptive pathway to allow the passage of teeth. Among these changes, an accentuated cell death by apoptosis and reduction in the blood vessel profiles were reported during the eruption of rat molars [10].

Therefore, the tooth eruption leads to a complex series of structural changes that occur in an extremely coordinated way avoiding an inflammatory reaction, which could compromise the integrity of dental tissues. Matrix metalloproteinases (MMPs) constitute a family of zinc-dependent endopeptidase responsible for the degradation of extracellular matrix (ECM) components [15]. MMPs are necessary for the development and maintenance of tissues and/or organs, exerting an important role in the tissue turnover/remodelling [15,16]. Moreover, MMPs are also detected under pathological conditions, cleaving the ECM components and leading to tissue degradation [17,18,19]. In rat molars, an enhanced MMP-9 immunoexpression was found in the lamina propria during the mucosal penetration stage of the tooth eruption [9]. As MMP-9 (Gelatinase B) cleaves denatured collagen, in particular, type IV collagen of the basal lamina, as well as degrades amorphous components of the ECM [20,21], the high immunoexpression of this metalloproteinase in the lamina propria was associated with tissue degradation and remodelling necessary for the establishment of the eruptive pathway [9]. MMP-1, other member of the MMPs superfamily, is a neutral proteinase that cleave native fibrillar collagens and, therefore, this MMP plays a key role in the degradation of the collagenous matrix [22,23]. MMP-1 can be released by several cells, including fibroblasts, macrophages and endothelial cells [19,24].

Delayed tooth eruption has been demonstrated in membrane type-1 matrix metalloproteinase deficient mice. In normal animals at 18–20 days of development, the first molars are already erupted. However, in the type-1 matrix metalloproteinase deficient mice, the molars show crowns with normal dentin and enamel, but the roots are truncated and the teeth are not erupted [25]. Another study in mice model with hyperplastic dental follicle (HDFs) showed reduced expression of MMPs (MMP-1 and MMP-3), whereas the expression of tissue inhibitor of metalloproteinases (TIMPs) and collagen increased, leading to thickening of fibrous components in HDFs. These results indicate that an appropriate control of the connective tissue remodelling is essential for the normal tooth eruption, and that MMPs, including MMP-1 and MMP-3, play important role during tooth eruption [26].

The degradation and remodelling of the eruptive pathway may also involve the phagocytosis and intracellular digestion processes. After the action of MMPs in the extracellular space, the remnants of the ECM components may be engulfed and digested by phagocytic cells. Although professional phagocytic cells such as macrophages exhibit high acid phosphatase content inside lysosomes [27], it has been shown that, in certain circumstances, ECM components [28,29] and apoptotic bodies can also be engulfed by fibroblasts [10,30]. Furthermore, the acid phosphatase activity inside phagocytic vacuoles of fibroblasts reinforces the concept that these cells can also act as phagocytic cells [28,29]. It has been described at least six types of acid phosphatase (ACP1, ACP2, ACP3, ACP4, ACP5, and ACP6) [31,32]. ACP2 is the lysosomal acid phosphatase, which degrades the intracellular collagen and plays an important role in tissue remodelling [29,33].

Few studies have focused on the importance of collagen remodelling for the tooth eruption, and the understanding of the specific molecular mechanisms underlying normal eruption is essential for the clinical diagnosis of eruptive disturbs. Considering that the lamina propria is a loose connective tissue containing mainly fibroblasts and macrophages intermingled with collagen fibres (predominantly types I and III) and amorphous substance [9,34,35] and the essential function of the MMP-1 in the cleavage of ECM components, we hypothesize that MMP-1 and acid phosphatase could have a role in the degradation of the eruptive pathway. Therefore, here, we evaluated the number of MMP-1-immunolabelled cells in the lamina propria and correlated this parameter with collagen content at the different eruptive phases. We also investigated whether acid phosphatase is involved in the degradation process of the lamina propria during tooth eruption.

## 2. Materials and Methods

Animal treatment was performed in accordance with Brazilian animal care and national laws on animal use. Our research protocol was authorized by the Ethical Committee for Animal Research of the São Paulo State University, Brazil (CEUA, Dental School-UNESP, Araraquara, protocol number 13/2013 approved on 14 June 2013).

Forty Holtzman postnatal male rats (*Rattus norvegicus albinus*) were divided into four groups (*n* = 10) according to their age: 9-, 11-, 13-, and 16-day-old. At these ages, the teeth are at specific eruptive phases. Therefore, the first molar germs of 9- and 11-day-old rats are at the intraosseous phase, whereas the first molars of 13- and 16-day-old rats are at the mucosal penetration phase [9,10]. The animals were housed in polypropylene cages that were filled with a layer of white pine shavings. During the experiment, one mother plus five male pups were housed per cage in a room with controlled temperature (23 ± 2 °C) and humidity (55 ± 10%). Rats were maintained under a 12:12 light/dark cycle with light onset at 07:00 h. Standardized chow (Guabi Rat Chow, Paulinia, SP, Brazil) and water were provided ad libitum.

The rats were killed by an overdose of ketamine hydrochloride and xylazine hydrochloride, decapitated and the upper maxillae were removed. The fragments of the maxilla from 5 rats per group were fixed and processed for light microscopy and molecular analysis (Western blot), whereas the fragments of maxilla from the other 5 rats were fixed and processed for transmission electron microscopy.

Using a stereoscopic microscope (Wild M7; Wild Heerbrugg, Heerbrugg, Switzerland), fragments of maxilla containing the first right molars were removed and placed in the fixative solution for light microscopy while the left maxillae were used for molecular analysis.

### 2.1. Light Microscopy

The fragments of maxilla containing the first right molar germs were fixed for 48 h at room temperature in 4% formaldehyde (freshly prepared from paraformaldehyde) buffered at pH 7.2 with 0.1 M sodium phosphate. After decalcification for 10 days in a 7% solution of EDTA (ethylenediaminetetraacetic acid) containing 0.5% formaldehyde (4), buffered at pH 7.2 with 0.1 M sodium phosphate-saline (PBS), the fragments of maxilla were dehydrated in graded concentrations of ethanol and embedded in paraffin. From each maxilla, fifty sagittal 6 µm-thick sections were collected onto slides. Five non-serial sections were stained with Carazzi’s haematoxylin and eosin (HE) whereas three non-serial sections were subjected to picrosirius-red method to estimate the collagen content. Other sections were adhered to silanized slides and submitted to immunohistochemistry for detection of MMP-1 and acid phosphatase (ACP-2).

### 2.2. Collagen Content Measurement in the Eruptive Pathway

For the collagen content estimation in the lamina propria of the eruptive pathway, the sections were submitted to the picrosirius-red method and analyzed under polarized light [36].

In each rat, three non-serial picrosirius-red-stained sections containing the lamina propria of the first molar were captured under polarized light at ×695 magnification; the smallest distance between the sections was 100 µm. In each section, three standardized fields were captured using an Olympus camera (DP-71, Olympus, Tokyo, Japan) attached to a light microscope (BX51, Olympus, Tokyo, Japan) at ×695 magnification, totalling a standardized area of 0.27 mm^2^ per section.

The birefringent collagen frequency was estimated using the following hue definition: red/orange 2–38 and 230–256, yellow 39–51 and green 52–128 [37]. The collagen content was calculated as a percentage of the area of each image (expressed in pixels) using ImageJ^®^ (NIH) as previously described [38]. Images were loaded and the hues were isolated using the hue histogram filter available in “Threshold Colour”. A black/white picture was created in which black pixels represented the hue in analysis (red/orange e.g.,) and the white pixels were the remaining hues. Total of black pixels was obtained and the percentage of birefringent collagen was calculated in the total area [39].

### 2.3. Immunohistochemical Detection of MMP-1

To unmask the antigenic sites, deparaffinized sections were immersed in 10 mM sodium citrate buffer pH 6.0, and placed into a microwave oven at 90–94 °C for 30 min. After a cooling-off period, the endogenous peroxidase was blocked with 3% hydrogen peroxide for 20 min. The slides were washed in 0.1 M PBS pH 7.2 and incubated for 30 min with 2% bovine serum albumin (BSA, Sigma-Aldrich Chemie, Munich, Germany). The sections were incubated overnight in a humidified chamber at 4 °C with mouse anti-MMP-1 primary antibody (MAB901; R&D System, Minneapolis, MN, USA), diluted 1:400. Sections were washed with PBS and the immunoreaction was detected by the Labelled Streptavidin-Biotin system (LSAB-plus Kit; DAKO Corporation, Carpinteria, CA, USA). The sections were incubated for 30 min at room temperature with a multi-link solution containing biotinylated anti-mouse/rabbit/goat secondary antibodies. After washing with PBS, the sections were incubated with the streptavidin-peroxidase complex for 30 min at room temperature. The sections were washed with PBS and the immunoreaction was revealed by 3,3′-diaminobenzidine (DAB-BiocareMedical, Concord, CA, USA); the sections were counterstained with Carazzi’s haematoxylin. For negative controls, the sections were incubated in non-immune serum (Sigma-Aldrich Chemie, Munich, Germany) instead of primary antibody.

### 2.4. Numerical Density of MMP-1-Immunolabeled Cells

Two non-serial sections exhibiting the lamina propria of the eruptive pathway of the first molar from each animal were used. The shortest distance between the sections was 100 µm. In each section, three standardized fields (0.09 mm^2^ each field) were captured using an Olympus camera (DP-71, Olympus, Tokyo, Japan) attached to a light microscope (BX51, Olympus, Tokyo, Japan) at ×695 magnification. The number of immunolabelled cells (brown-yellow colour) was computed by one blinded and calibrated examiner using an image analysis system (Image Pro-Express 6.0, Olympus, Silver Spring, MD, USA). The number of MMP-1-positive cells/mm^2^ of the lamina propria per animal was calculated [10,19].

### 2.5. Immunohistochemical Detection of Acid Phosphatase (ACP-2)

For antigen retrieval, deparaffinized sections were immersed in distilled water containing 0.1% CaCl_2_ and 0.5% trypsin for 1 h and 30 min at 37 °C. After the inactivation of endogenous peroxidase in 3% hydrogen peroxide for 20 min, the sections were washed in 0.1 M PBS pH 7.2 and incubated for 30 min. with 2% BSA (Sigma-Aldrich, St. Louis, MO, USA). Subsequently, the sections were incubated overnight in a humidified chamber at 4 °C with mouse anti-acid phosphatase primary antibody (sc-100344; ACP-2-Santa Cruz Biotechnology, Inc^®^, Santa Cruz, CA, USA) diluted 1:100. Sections were washed with PBS and the immunoreaction was amplified using the Labelled Streptavidin-Biotin system (LSAB-plus Kit; DAKO Corporation, Carpinteria, CA, USA), as described above. Peroxidase activity was revealed by 3,3′-diaminobenzidine (DAB-BiocareMedical, Concord, CA, USA) and the sections were counterstained with Carazzi’s haematoxylin. As negative controls, the sections were incubated in non-immune serum (Sigma-Aldrich Chemie, Munich, Germany) instead of primary antibody.

### 2.6. Protein Extraction and Western Blot for MMP-1 and ACP-2

For molecular analysis, small fragments of the eruptive pathway were obtained from the 16-day-old rats. Using a scalpel (Fibra Cirúrgica, Joinvile, Brazil), the oral mucosa overlaying the upper first molars was carefully removed with the help of a stereoscopic microscope (Wild M7; Wild Heerbrugg, Switzerland) at ×60. The small fragments of oral mucosa of the eruptive pathway were frozen at −80 °C for Western blot.

Frozen fragments of oral mucosa of the eruptive pathway were homogenized directly into lysis buffer (50 mM Tris pH 8.0, 150 mM NaCl, 1 mM EDTA, 10% glycerol, 1% Triton X-100, 1 mM phenylmethylsulfonyl fluoride (PMSF)), containing 5 ng/mL of each of the following protease inhibitors: Pepstatin, Leupeptin, Aprotinin, Antipain, and Chymostatin (Sigma-Aldrich^®^; P8340). The crude extracts were clarified by centrifugation at 10,000 rpm for 20 min and 4 °C, and the supernatant was collected. Protein concentration was determined using Bradford assay (Sigma-Aldrich^®^; B6916), and Western blot for MMP-1 and ACP-2 detection was performed. The same amount of proteins (20 µg) was mixed with an equivalent volume of Laemmli buffer and heated at 95 °C for 5 min. before electrophoresis. The samples were separated in sodium dodecyl sulfate polyacrylamide gels (SDS-PAGE; 12% polyacrylamide), and then transferred to a nitrocellulose membrane (GE Healthcare^®^, Chicago, IL, USA). The membrane was treated with blocking solution containing 5% non-fat milk, and then incubated overnight at 4 °C with mouse anti-MMP-1 primary antibody (MAB901; 1:400; R&D System, Minneapolis, MN, USA) or with mouse anti-acid phosphatase primary antibody (sc-100344; 1:100; ACP2, Santa Cruz Biotechnology, Inc^®^, Santa Cruz, CA, USA) diluted in PBS containing 0.05% Tween 20 (PBS/T). After washing, the membranes were incubated for 1 h with anti-rabbit (Sigma-Aldrich, St. Louis, MO, USA) or anti-mouse (Sigma-Aldrich, USA, A9044) peroxidase antibodies diluted with PBS/T solution (1:1250). The reactions were detected using an enhanced chemiluminescence system (ECL) and the bands were visualized using a digital documentation system (GelDoc XR, Bio-Rad Laboratories, Hercules, CA, USA). As loading control, the membranes were probed with rabbit anti-actin antibody (1:8000; Sigma-Aldrich, Sigma-Aldrich, St. Louis, MO, USA). The assays were performed in triplicate for each protein.

### 2.7. Transmission Electron Microscopy

For ultrastructural analysis, five right maxilla per group were used. The fragments of maxilla containing the lamina propria overlaying the first molar germs were fixed for 16 h in a solution of 4% glutaraldehyde and 4% formaldehyde buffered at pH 7.2 with 0.1 M sodium cacodylate [40]. After decalcification for 10 days in a solution of 7% EDTA buffered at pH 7.2 in 0.1 M sodium cacodylate, the specimens were postfixed in sodium cacodylate-buffered 1% osmium tetroxide at pH 7.2 for 1.5 h. Subsequently, the specimens were washed in distilled water and immersed in 2% aqueous uranyl acetate for 2 h. After washing in distilled water, the specimens were dehydrated in graded concentrations of ethanol, treated with propylene oxide and then embedded in Araldite. Semithin sections stained with aqueous solution of 1% toluidine blue and 1% sodium borate were examined under light microscope, and suitable regions were carefully selected for trimming of the blocks. Ultrathin sections were collected onto grids, stained in alcoholic 2% uranyl acetate and in lead citrate solution and examined under a transmission electron microscope (Tecnai G2 Spirit, FEI Company, Hillsboro, OR, USA).

### 2.8. Ultrastructural Localization of Acid Phosphatase Activity

For ultrastructural localization of acid phosphatase, five left maxilla per group were used. The oral mucosa overlaying the upper first molar germs was removed with the help of a stereoscopic microscope (Wild M7; Wild Heerbrugg, Switzerland) at ×60 magnification. Small fragments of the oral mucosa containing the lamina propria of the eruptive pathway were fixed for 6 h in a solution of 2% formaldehyde and 0.5% glutaraldehyde buffered at pH 7.2 with 0.1 M sodium cacodylate. After fixation, the specimens were washed in sodium cacodylate buffer and incubated in a medium prepared by dissolving 20 mM lead nitrate (Merck, Darmstadt-DE, Germany) in 0.1 M sodium acetate buffer (pH 5.0) followed by addition of 10 mM sodium β-glycerophosphate (Merck, Darmstadt-DE), as described by Barka [41]. After addition of magnesium chloride, the solution was filtered and the specimens were incubated for 2 h at 37 °C. As control of specificity, some specimens were incubated in substrate-free medium (without β-glycerophosphate). Subsequently, the specimens were fixed for 2 h in 3% glutaraldehyde buffered at pH 7.2 with 0.1 M sodium cacodylate at 4 °C. Then, the specimens were washed and immersed in 1% osmium tetroxide for 1 h. After washing, the specimens were dehydrated in graded concentrations of ethanol, treated with propylene oxide and then embedded in Araldite. Ultrathin sections were examined with Tecnai electron microscope.

### 2.9. Statistical Analysis

The statistical analyses were performed using Statistical Sigma Stat 3.2 software (Jandel Scientific, Sausalito, CA, USA). The differences in the numerical density of MMP-1-immunolabelled cells and birefringent collagen content were statistically analyzed among all groups (9-, 11-, 13- and 16-day-old rats). One-way ANOVA was used for the analysis of variance followed by Tukey post-hoc test. The significance level was set at *p* ≤ 0.05.

The correlation between numerical density of MMP-1-immunolabelled cells and collagen content was evaluated by Pearson product-moment coefficient. The significance level considered was *p* ≤ 0.05.

## 3. Results

### 3.1. Morphological Findings and Content of Birefringent Collagen

**Figure 1:**
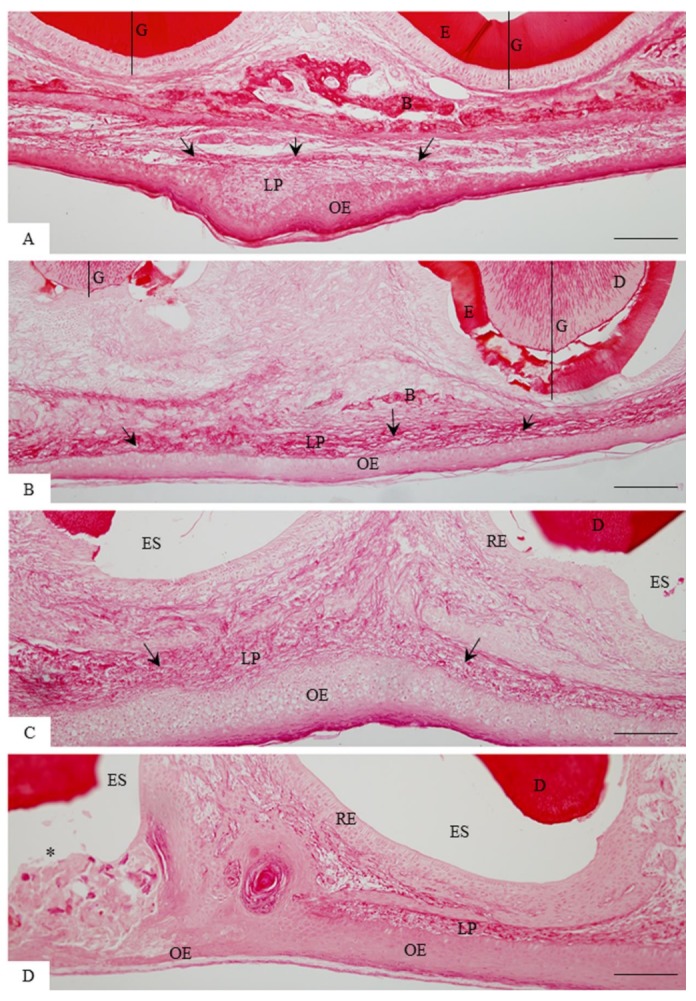
Light micrographs of sagittal sections of maxilla showing portions of the eruptive pathway of the first molar of 9- (**A**), 11- (**B**), 13- (**C**), and 16-day-old (**D**) rats. In **A** and **B**, the lamina propria (LP) contains bundles of collagen fibres (arrows). Note that in the 9-day-old rat (**A**), a continuous layer of bone trabeculae (**B**) is observed between the tooth germ (G) and oral epithelium (OE). In **B**, a thin bone trabecula (**B**) is present in the area between the molar cusps. In **C** and **D**, the delicate collagen fibres (arrows) are irregularly distributed in the thin lamina propria (LP). In **D**, a cusp tip (asterisk) is passing through the oral epithelium (OE). D, dentine; E, enamel matrix; ES, enamel space; RE, reduced enamel epithelium. Stained with picrosirius. Bars: 50 µm.

**Figure 2:**
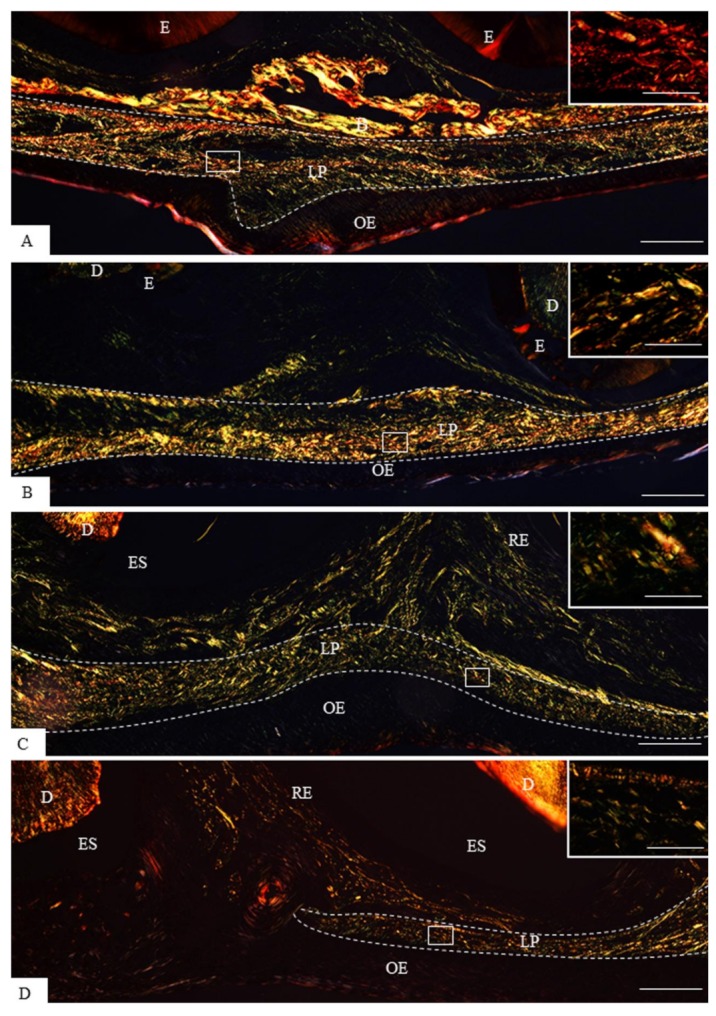
Light micrographs of sagittal sections of maxilla showing portions of the eruptive pathway of the first molar of 9- (**A**), 11- (**B**), 13- (**C**), and 16-day-old (**D**) rats, subjected to the picrosirius-red and analyzed under polarized light. The hatched lines delimit the lamina propria (LP) of the eruptive pathway. Irregularly arranged birefringent collagen fibres are distributed throughout the lamina propria (LP) of 9- (**A**) and 11-day-old (**B**) rats. The lamina propria (LP) contains mainly bundles of birefringent collagen exhibiting red (**A**, inset) and yellow (**B**, inset) colours. **C** and **D**—few birefringent collagen fibres exhibiting the hue varying from yellow to green are present in the lamina propria (LP) of 13- and 16-day-old rats. The insets, outlined areas in **C** and **D**, show thin bundles of birefringent collagen fibres. D, dentine; E, enamel matrix; B, bone trabeculae; OE, oral epithelium; RE, reduced enamel epithelium. Bars: 50 µm and 5 µm (insets).

**Figure 3:**
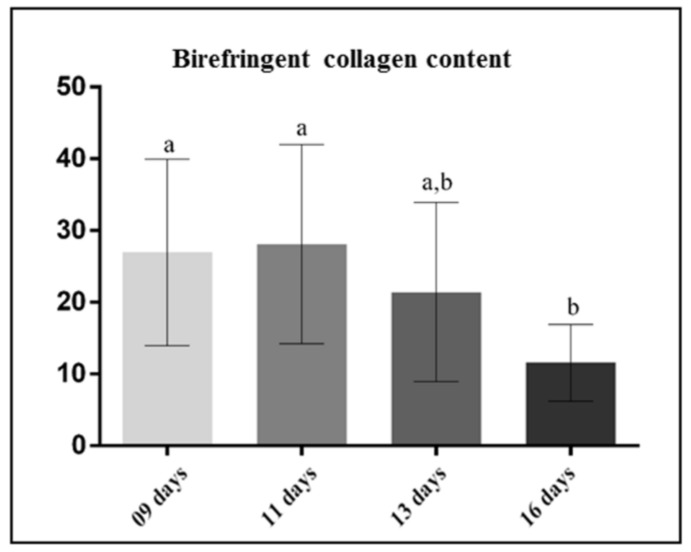
Birefringent collagen content (percentage) in the lamina propria of the eruptive pathway from 9-, 11-, 13-, and 16-day-old rats. Significant reduction in the collagen content is observed in 16-day-old rats in comparison to 9- and 11-day-old rats. Significant difference is not observed between 13- and 16-day-old rats. One way ANOVA and the Tukey post-hoc test (*p* ≤ 0.05). Statistically significant difference among groups is indicated by different letters.

The analysis of sagittal sections of maxillae revealed changes in the arrangement and in the content of collagen of the lamina propria of the eruptive pathway in the rats at different ages (Figure 1 and Figure 2). In the 9- and 11-day-old rats, evident bundles of collagen fibres were distributed throughout lamina propria (Figure 1A,B) while scarce collagen fibres were present in the thin lamina propria of 13- and 16-day-old rats (Figure 1C,D). Under polarized light, the sections subjected to the picrosirius-red staining revealed a different pattern of birefringent colours in the lamina propria in accordance with the eruptive phase of first molars (Figure 2). In the 9- and 11-day-old rats, the lamina propria showed an evident continuous layer of birefringent bundles of collagen with the hue varying from red/orange to yellow colour (Figure 2A,B; insets). Otherwise, few and thin birefringent collagen content was observed in the eruptive pathway of 13- and 16-day-old rats (Figure 2C,D). At these time points, the thin bundles of collagen material exhibited predominantly yellow and green colours (Figure 2C,D, insets). Quantitative analysis revealed a significant decrease in the birefringent collagen content in the lamina propria of 16-day-old rats in comparison to 9- (*p* = 0.007) and 11- (*p* = 0.012) day-old rats (Figure 3).

**Figure 4:**
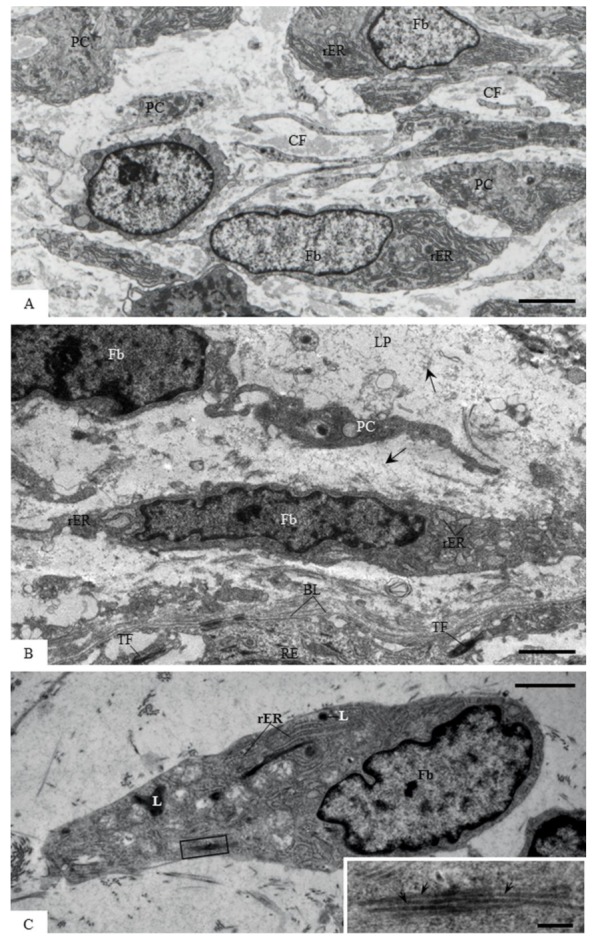
Electron micrographs of portions of lamina propria of the eruptive pathway of first molars of 9- (**A**) and 16- (**B** and **C**) day-old rats. In **A**—fibroblasts (Fb) exhibiting several rough endoplasmic reticulum profiles (rER) in their large cytoplasm are surrounded by numerous collagen fibrils (CF). PC, cell portions. Bar: 5 µm. In **B**—Fibroblasts (Fb) containing few rough endoplasmic reticulum profiles (rER) in the scarce cytoplasm are surrounded by granular material (arrows) in the extracellular matrix. PC, cell portion; RE portion of the reduced enamel epithelium; TF, bundles of tonofilaments; BL, basal lamina. Bar: 5 µm. **C**—a polarized fibroblast (Fb) with irregular nucleus exhibits collagen fibrils (outlined area) apparently internalized in the large cytoplasm. The inset of the outlined area shows the profiles of banded collagen fibrils (arrows). rER, rough endoplasmic reticulum profiles; L, lysosomes. Bars: 3 µm and 0.4 µm (inset).

Ultrastructural analysis of the lamina propria at the initial phases of tooth eruption exhibited elongated fibroblasts with well-developed rough endoplasmic reticulum and Golgi complex in their large cytoplasm; several bundles of collagen fibrils in the extracellular matrix were observed (Figure 4A). At the advanced stage of tooth eruption (13- and 16-day-old rats), the extracellular matrix showed a granular material and scarce collagen fibrils (Figure 4B). Moreover, fibroblasts exhibiting collagen fibrils in their cytoplasm were also observed (Figure 4C).

### 3.2. MMP-1 Immunoexpression in the Lamina Propria

**Figure 5:**
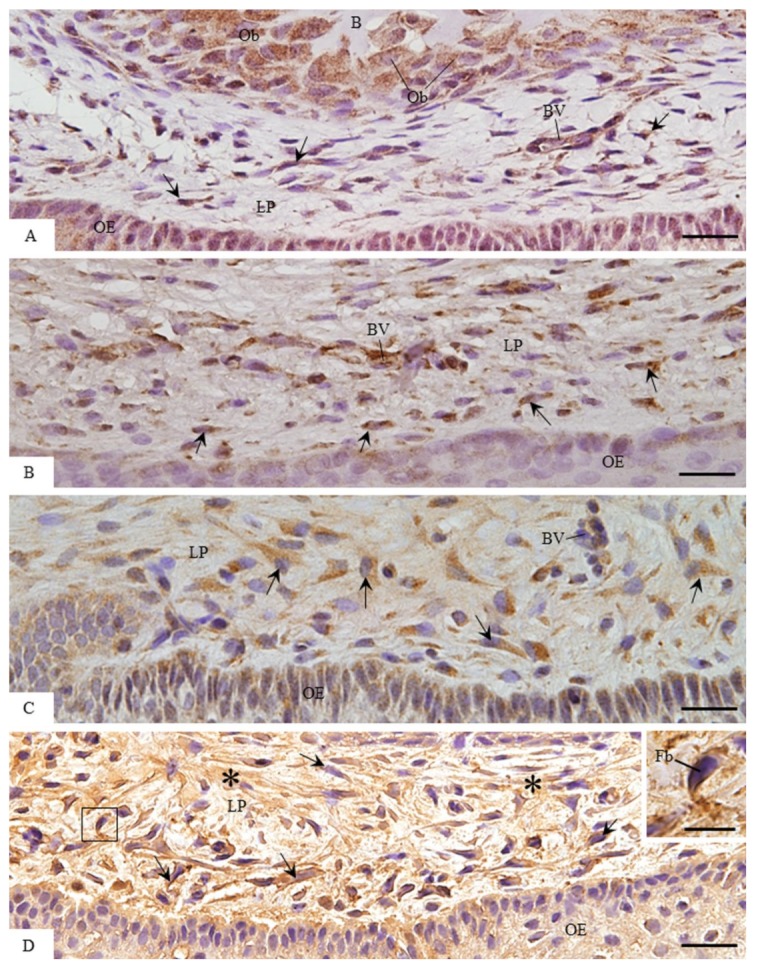
Light micrographs of portions of oral mucosa of the eruptive pathway of first molars of 9- (**A**), 11- (**B**), 13- (**C**), and 16-day-old (**D**) rats. The sections were subjected to the immunohistochemistry for detection of MMP-1 (brown-yellow colour) and counterstained with haematoxylin. Immunolabelled cells (arrows) are observed in the lamina propria (LP) at different stages of tooth eruption. High magnification (inset), outlined area of **D**, shows immunostained fibroblast cytoplasm (Fb). Note an enhanced immunolabelling in the lamina propria (LP) of 13- and 16-day-old rats (**C** and **D**); immunolabelling is observed in the extracellular matrix (asterisks). Ob, osteoblasts; B, bone matrix; OE, oral epithelium; BV, blood vessel. Bars: 20 µm and 6 µm (inset).

**Figure 6:**
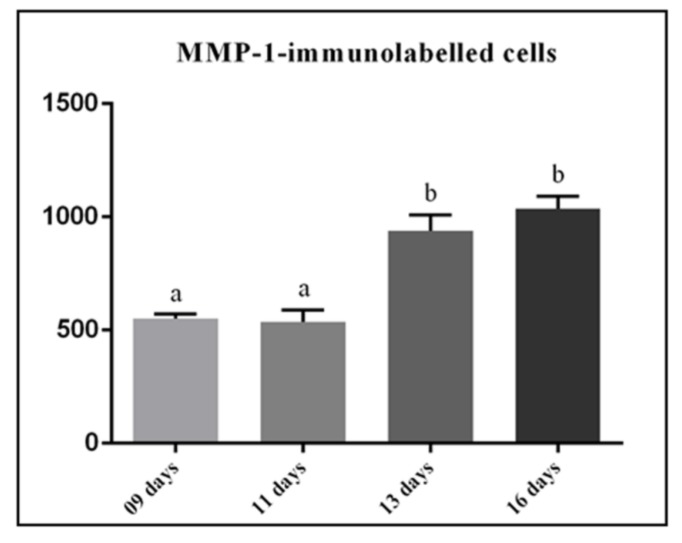
The number of MMP-1-immunolabelled cells per mm^2^ of lamina propria of the eruptive pathway from 9-, 11-, 13-, and 16-day-old rats. Significant increase of immunolabelled cells is observed in 13- and 16-day-old rats in comparison to 9- and 11-day-old rats. One way ANOVA and the Tukey post-hoc test (*p* ≤ 0.05). Statistically significant difference among groups is indicated by different letters.

In all the groups, strong immunolabelling in the cytoplasm (brown-yellow colour) of fibroblasts was observed in the lamina propria (Figure 5). However, an enhanced immunoexpression was evident in the lamina propria of 13- and 16-day-old rats (Figure 5C,D). MMP-1-immunoreaction was also detected in the macrophages and endothelial cells (Figure 5A,B). Moreover, conspicuous immunolabelling was seen in the osteoblasts next to the bone trabeculae located in the eruptive pathway of 9-day-old rats (Figure 5A). In the maxilla sections incubated in the non-immune serum (negative control), no MMP-1-immunolabelled cell was observed (data not shown). According to Figure 6, no significant difference was found in the number of MMP-1-immunolabelled cells/mm^2^ between 9- and 11-day-old rats (*p* = 0.998) as well as between 13- and 16-day-old rats (*p* = 0.56). On the other hand, a significant increase (*p* < 0.001) in the immunolabelled cells was observed in the 13- and 16-day-old rats in comparison with the groups of 9- and 11-day-old rats.

A significant correlation (*p* ≤ 0.01) was observed between immunoexpression for MMP-1- and collagen content. Moreover, this analysis revealed that these parameters were inversely proportional (r = −0.614).

### 3.3. ACP-2 Immunoexpression in the Lamina Propria

**Figure 7:**
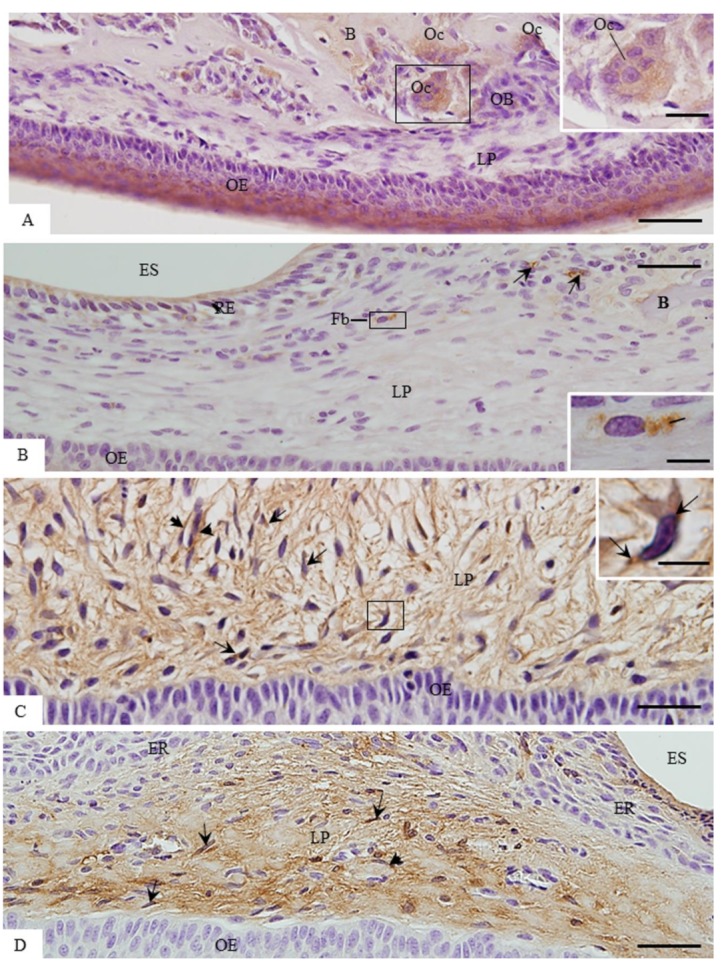
Light micrographs of portions of oral mucosa of the eruptive pathway of first molars subjected to the immunohistochemistry for detection of ACP-2 (brown-yellow colour) and counterstained with haematoxylin. In **A** (9-day-old rat) and **B** (11-day-old rat) scarce immunolabelled cells (arrows) are observed in the eruptive pathway. In **A**, ACP-2 immunolabelled multinucleated osteoclasts (Oc) are present in the bone (**B**) surface. The inset, outlined area in **A**, shows immunolabelled osteoclast (Oc) cytoplasm. LP, lamina propria. Bars: 40 µm and 15 µm (inset). In **B**, an immunolabelled fibroblast (Fb) is observed between the lamina propria (LP) and reduced enamel epithelium (RE). Immunolabelled cells (arrows) are observed next to the bone (B) surface. The inset, outlined area, shows conspicuous immunostaining in the fibroblast cytoplasm (Fb). OE, oral epithelium; ES, enamel space. Bars: 40 µm and 6 µm (inset). In **C** (13-day-old rat) and **D** (16-day-old rat), several immunolabelled fibroblasts (arrows) are seen in the lamina propria (LP). In the inset, outlined area of **C**, strong immunostaining is observed in the cytoplasm of a fibroblast (arrows). ACP-2-positive immunolabelling is observed in the endothelial cells (arrowheads). ES, enamel space; ER, reduced enamel epithelium; OE, oral epithelium. Bars: 40 µm (**C** and **D**) and 6 µm (inset).

Sections subjected to immunohistochemistry for ACP-2 detection revealed a distinct immunolabelling pattern according to the stage of tooth eruption (Figure 7). In the 9- and 11-day-old rats, scarce or no immunolabelled cells were observed in the lamina propria (Figure 7A,B). In the 9-day-old rats, immunolabelling in the osteoclast cytoplasm was often observed (Figure 7A). Occasionally, immunoreaction was also found in the fibroblasts of the eruptive pathway, located next to the dental follicle (Figure 7B). On the other hand, conspicuous immunolabelling was often observed in the cytoplasm of fibroblasts and macrophages in the groups of 13- and 16-day-old rats (Figure 7C,D). In addition, immunostaining was observed in the endothelial cells of the blood vessels in the eruptive pathway (Figure 7C,D). In the maxilla sections used as negative controls, immunolabelled cells were not found (data not shown).

### 3.4. Detection of MMP-1 and ACP-2 by Western Blot

**Figure 8:**
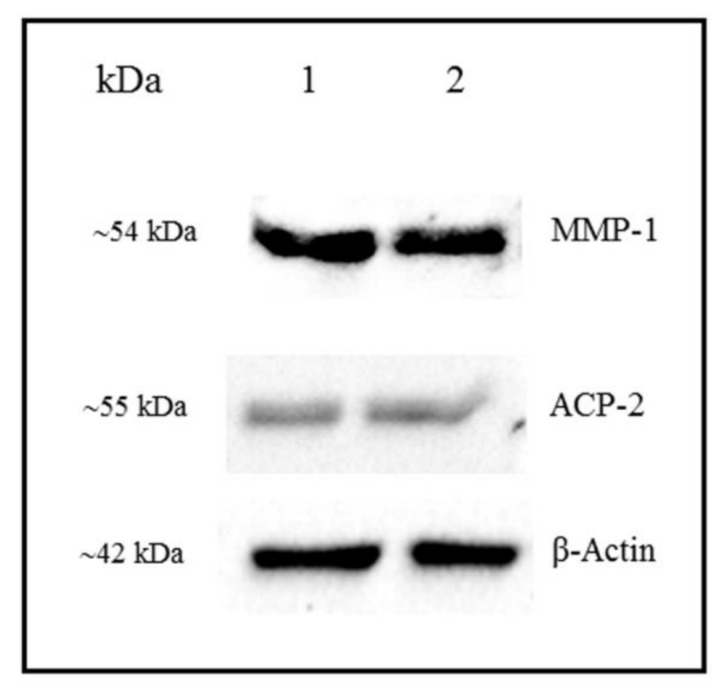
Western blot of MMP-1 and ACP-2 from extracts of oral mucosa of the eruptive pathway obtained from two 16-day-old rats. Bands at 54 kDa and 55 KDa levels, corresponding to MMP-1 and ACP-2 molecular weights, respectively, are observed. Actin bands (~42 KDa) are also seen.

As shown in Figure 8, Western blot analyses of protein extracts from oral mucosa of the eruptive pathway demonstrated evident MMP-1 and ACP2 immunoreactive bands at ~54 KDa and ~55 KDa, respectively, confirming the specificity of the antibodies to rat tissues. A strong band at 42 KDa, corresponding to actin, was also observed (Figure 8).

### 3.5. Ultrastructural Localization of Acid Phosphatase Activity

**Figure 9:**
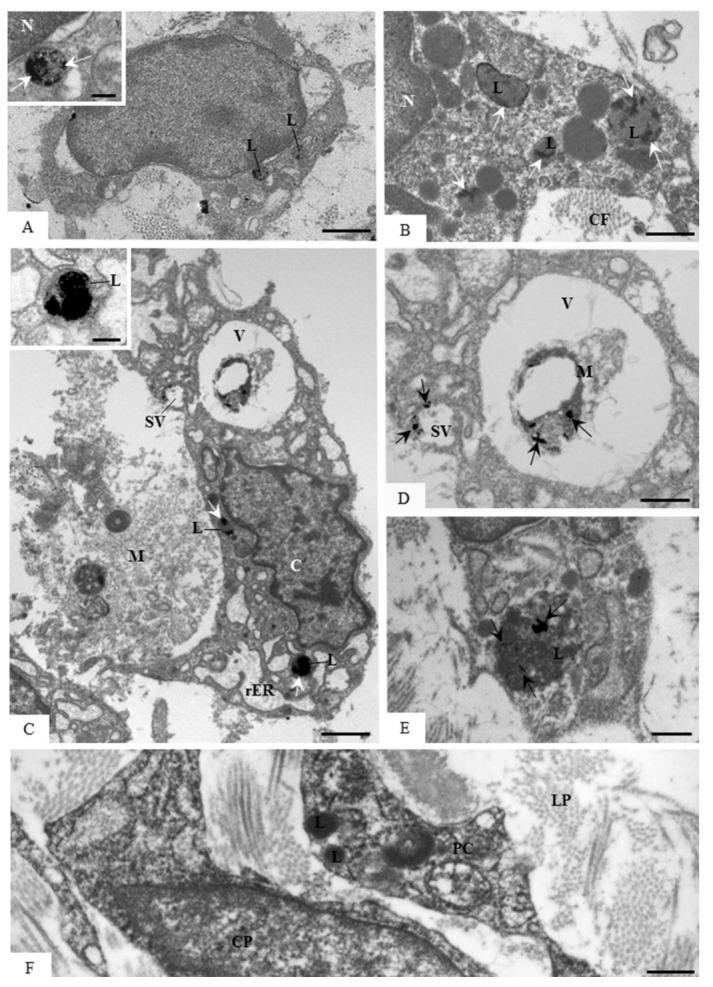
Electron micrographs of portions of lamina propria of the eruptive pathway of first molars of 9- (**A**), 13- (**B** and **F**), 16- (**C**–**E**) day-old rats. **A**–**E**—portions of the eruptive pathway of first molars incubated for acid phosphatase reaction. **A**—a round-shaped cell exhibits electron-opaque deposits in the lysosomes (L). In high magnification (inset), conspicuous electron-opaque deposits (arrows)—reaction product of the acid phosphatase activity—is irregularly distributed throughout the lysosome. N, nucleus. Bars: 5 µm and 0.2 µm (inset). In **B**, several lysosomes (L) exhibiting electron-opaque deposits (arrows) are observed in the cytoplasm of a macrophage. N, nucleus; CF, collagen fibrils. Bar: 1.5 µm. **C**—an irregular cell (C) with large vacuole (V) is surrounding partially a heterogeneous material (M). Electron-opaque deposits (arrows) are observed in the lysosomes (L) and in the periphery of a small vacuole (SV). The inset shows conspicuous electron-opaque deposits almost entirely filling a lysosome (L). rER, rough endoplasmic reticulum. Bars: 4 µm and 0.3 µm (inset). **D**—high magnification of the large vacuole (V) observed in the superior portion of the Figure 9C. Fine products of the reaction to acid phosphatase (arrows) are observed on the partially digested material (M). Granular electron-opaque deposits (arrows) are also seen in the periphery of a small vacuole (SV). Bar: 1 µm. Figure 9**E**—a large lysosome (L) exhibiting acid phosphatase-positive deposits (arrows) intermingled with heterogeneous material is present in the fibroblast. Bar: 0.5 µm. **F**—ultrathin section of a portion of the eruptive pathway incubated in substrate-free medium (negative control). No reaction product is observed in the lysosome (L) of cellular portions (CP) in the lamina propria (LP). Bar: 1.5 µm.

The ultrathin sections of the specimens incubated in the medium containing β-glycerophosphate revealed electron-opaque deposits of reaction product, i.e., acid phosphatase activity (Figure 9). A distinct pattern of acid phosphatase reaction was observed in the cells of the lamina propria according to the eruptive phase. Few cells exhibiting deposits of reaction product in the lysosomes were observed in the lamina propria during the intraosseous eruptive phase (Figure 9A) whereas conspicuous acid phosphatase activity was seen in the cells during the mucosal penetration phase of tooth eruption (Figure 9B–E). In some irregular-shaped cells—macrophage-like cells, phosphatase acid activity was also seen in the large vacuoles containing partially degraded material (Figure 9C,D). No reaction product was seen in the specimens incubated without β-glycerophosphate (Figure 9F).

## 4. Discussion

**Figure 10:**
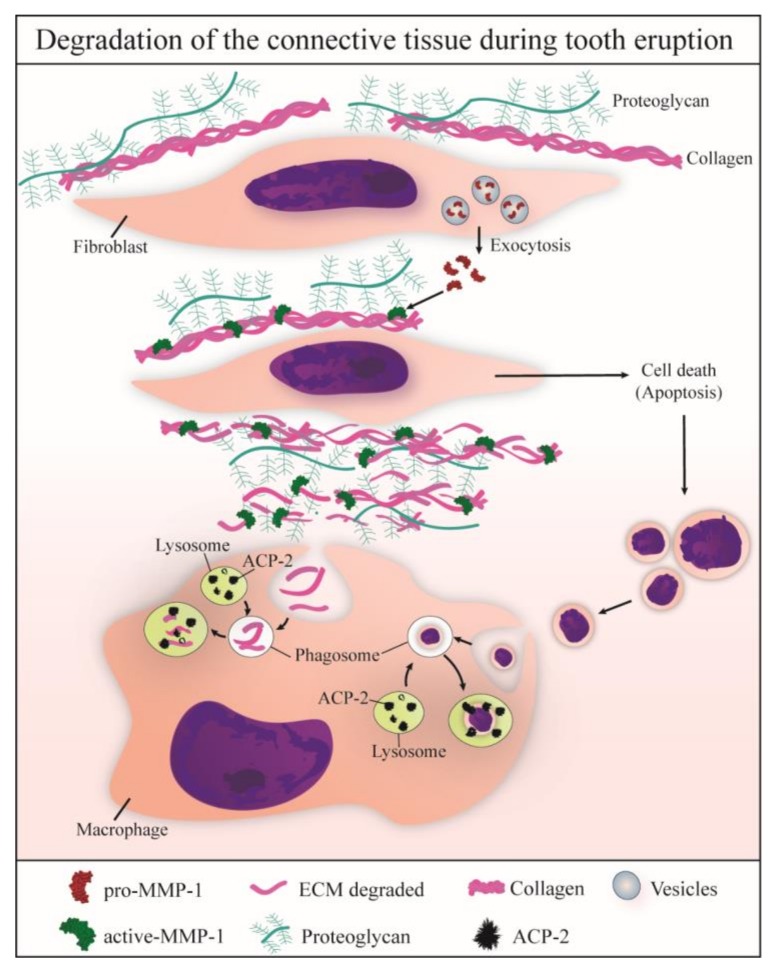
Schematic figure showing some mechanisms involved in the degradation of ECM of eruptive pathway during the normal tooth eruption. Fibroblasts produce and release pro-MMP-1, which is activated in the extracellular microenvironment and becomes active-MMP-1. This enzyme degrades collagen fibrils surrounding fibroblasts and other cells, which undergo apoptosis. Either collagen fragments or apoptotic bodies are engulfed by neighbouring macrophages. Phagosomes containing collagen and/or apoptotic bodies are fused with lysosomes containing lysosomal enzymes, such as ACP-2, which allows the intracellular digestion of these eruptive pathway components.

The significant increase in the MMP-1 immunoexpression in parallel to accentuated reduction in the birefringent collagen content during the mucosal penetration phase of tooth eruption indicates that MMP-1 is involved in the degradation of the extracellular matrix (ECM) components of the lamina propria of the eruptive pathway. Our findings also indicate that ECM components degraded by MMP-1 are engulfed by macrophages and fibroblasts of the lamina propria and digested by acid phosphatase activity within these cells, as summarized in Figure 10.

MMP-1 has an important participation during development as well as in the maintenance, repair and regeneration of several tissues and organs. During growth of fly Mmp-1 mutants, tracheal tubes cannot expand and dilate properly to follow the larval growth and, consequently, they break from the stretching tension. This disturbance in tracheal tube growth is caused by failure of degradation of attachment components between cells and ECM due to lack of MMP-1 [42]. MMP-1 expression by keratinocytes at onset of healing seems to be essential to migration and orientation of these cells during reepithelialization [43]. When MMP-1 activity is blocked by TIMP-1, the apoptosis is inhibited indicating that this metalloproteinase also exerts a control under the cell survival [44]. On the other hand, overexpression of MMP-1 leads to tumour progression and metastasis [45]. Elevated MMP-1 expression has been associated with several tumours such as peritoneal metastasis in gastric cancer [46], colorectal cancer [47], and cutaneous melanoma cancer [47,48].

In the present study, the accentuated MMP-1 immunoexpression in association with the significant reduction in the birefringent collagen content indicates that this enzyme exerts an intense activity in the eruptive pathway mainly at the mucosal penetration stage. Moreover, the reduction in the birefringent content was accompanied by changes in the colour pattern exhibited by the birefringent collagen in the lamina propria. At the intraosseous eruptive phase (9- and 11-day-old rats), the thick bundles of collagen fibres exhibited predominantly red/orange and yellow colours whereas in the mucosal penetration phase (13- and 16-day-old rats), the yellow and green birefringence of thin collagen fibres were often seen. Although the birefringence colour is not useful to identify the molecular collagen nature [49,50], it has been suggested that birefringence colour reflects the collagen fibre diameter. Thus, the birefringence colours from red to orange, to yellow, to green correspond to decreasing fibre diameter [50,51]. Here, few thin collagen bundles exhibiting yellow or green birefringence were often observed at the advanced stage of tooth eruption, indicating that the thick bundles of fibres with red/orange or yellow birefringence present in the intraosseous stage were at least in part degraded during the establishment of the eruptive pathway. In fact, a significant increase in the immunoexpression of MMP-1 was detected in the eruptive pathway of 13- and 16-day-old rats. The specificity of the MMP-1 antibody to rat tissues was confirmed by Western blot, which revealed bands at ~54 KDa, corresponding to MMP-1 [15].

Our findings showed immunolabelling in the cytoplasm of fibroblasts and macrophages of the lamina propria at the different eruptive phases. It is known that MMP-1 is responsible for degradation of collagen type I, II, III, V, and XI [52]; this MMP is produced and released by different cell types, including fibroblasts, macrophages, and neutrophils [19,53,54]. Usually the MMPs are synthesized as inactive proenzymes and are activated by several factors such as FGF (fibroblast growing factor), TGF (transforming growing factor) and IL-1 (interleukin-1) in the extracellular matrix [55,56,57]. Moreover, tissue inhibitors of metalloproteinases (TIMPs) regulate the proteolytic activity of the MMPs, and the balance between MMP/TIMPs is responsible for extracellular matrix turnover [26,58]. Evidence indicates that MMPs have pivotal role in the tissue degradation of the eruptive pathway [9,26] and decreased expression of these enzymes may impair the tooth eruption and induces, for example, non-syndromic hyperplastic dental follicle [26]. Failure of tooth eruption and odontoma-like structures formation in the op/op (osteopetrotic) mouse were associated with intense disordered ECM remodelling [59], indicating that MMP/TIMP unbalance is responsible for tooth eruption disturbance and may lead to formation of tumour lesions.

During tooth eruption, an accentuated expression of IL-1 [56] and TNF-α [57] has been demonstrated in the dental follicle. Among several functions, these cytokines released by cells from the dental follicle stimulate the osteoclast formation and subsequent bone resorption during tooth eruption [11]. It is possible that these cytokines also promote the cleavage of the pro-MMP-1, activating this metalloproteinase in the eruptive pathway. Differences in the pattern of MMP-9 immunoexpression have also been reported in the eruptive pathway of rat molars; an enhanced immunoexpression has been seen at the mucosal penetration phase, particularly in the 16-day-old rats [9]. MMP-9 is responsible for degradation of amorphous components of the extracellular matrix and denatured type I collagen. Thus, the accentuated immunoexpression at the advanced stage of tooth eruption indicates that MMP-9 acts in the degradation of the lamina propria [9].

Our findings showed a significant increase in the MMP-1-immunolabelled cells over time, indicating that this enzyme may be responsible for collagen breakdown of the lamina propria (Figure 10), leading to reduction of the collagen content as well as changes in the collagen thickness at the mucosal penetration phase of the tooth eruption. This idea is supported by the significant correlation between MMP-1 immunoexpression and collagen content. In fact, the collagen content of the lamina propria reduced 57% from 9 days to 16 days, and this reduction was accompanied by an increase in the number of MMP-1-immunolabelled cells (89%) in the lamina propria. At advanced eruptive stage, the ECM showed an evident immunoreactivity for MMP-1, and the ultrastructural analysis revealed scarce collagen fibrils and granular material surrounding the cells in the lamina propria confirming that the extracellular matrix components are undergoing degradation. It is important to emphasize that at 16 days the first rat molars are passing through the oral mucosa and, therefore, structural changes are necessary in the lamina propria to allow the tooth eruption. Among these structural changes in the lamina propria, we have demonstrated an accentuated apoptosis index and reduction in the blood vessel profiles during mucosal penetration phase of the tooth eruption [10]. In the present study, the increase in the number of MMP-1-immunolabelled cells at the mucosal penetration stage, confirms the role of MMP-1 in the degradation of the eruptive pathway. Thus, the MMPs, including the MMP-1, exert a crucial role in the degradation of the eruptive pathway and the deficiency of these enzymes may impair tooth eruption. This hypothesis is reinforced by the fact that a marked decrease in the expression of MMP-1 and MMP-3 was detected in the thick fibrous connective tissue of hyperplastic dental follicle [26]. 

Our findings revealed immunolabelling in the endothelial cells suggesting a possible participation of MMP-1 in the microvasculature remodelling. In addition to the well-established role of MMP-1 in the ECM components breakdown, it has been reported that MMPs can also regulate the vascular proliferation [60,61,62]. Thus, it is conceivable to suggest that MMP-1 has also a role in the rearrangement of the vascular plexus since the involution of blood vessel in the eruptive pathway may be concomitant with vascular proliferation necessary for gingiva development during tooth eruption [10]. It is important to emphasize that a marked increase of blood vessels has been reported in opercular lesions in non-erupted human permanent molars [5]. Thus, there is a consensus regarding the fact that any disturbance in the breakdown of the oral mucosa of the eruptive pathway may delay the tooth eruption [63].

Regarding the ACP-2 immunohistochemical detection, scarce immunolabelled cells were observed in the lamina propria at the intraosseous eruptive phase, whereas an evident immunostaining was detected in the osteoclasts adjacent to the bone surface overlaying the developing tooth germ of 9- and 11-day-old rats. It is known that osteoclasts express high levels of acid phosphatases, including the tartrate-resistant acid phosphatase (TRAP), also named ACP5 [64,65,66], and the lysosomal acid phosphatase, ACP-2. ACP-2 is an important enzyme for lysosomal function, responsible for the hydrolysis of orthophosphoric monoesters to alcohol and phosphate [33]. Here, the monoclonal antibody raised against recombinant ACP-2 of human origin was reactive to rat tissues, as demonstrated by Western blot analysis, which revealed bands at ~55 KDa [33].

A conspicuous immunostaining for ACP-2 in the fibroblasts and macrophages observed in the 13- and 16-day-old rats indicates that these cells act in the degradation process of the lamina propria during the mucosal penetration phase of tooth eruption. Moreover, ACP-2 immunolabelling was also observed in the endothelial cells as also described in other tissues [67,68]. Here, the presence of ACP-2 in the endothelial cells, particularly at the advanced stages of tooth eruption, indicates that this enzyme is involved in the vascular remodelling, which is necessary during tooth eruption. The apoptosis of vascular cells together with the significant reduction in the blood vessel profiles in the lamina propria indicates that a rearrangement of the microvascular plexus occurs in the eruptive pathway at the mucosal penetration phase [10]. It is known that apoptotic bodies can be recognized and engulfed by neighbouring cells [10,30,40,63,69]. Therefore, it is possible that the ACP-2 immunoexpression in the endothelial cells may reflect an increase of phagocytosis and intracellular digestion of apoptotic vascular cells.

In the present study, the immunolocalization of ACP-2 was consistent with the ultrastructural localization of the acid phosphatase activity. The ultrathin sections from specimens incubated with β-glycerophosphate showed electron-opaque deposits irregularly distributed in the lysosomes of fibroblasts and macrophages, indicating ultrastructural features of reaction product of acid phosphatase [28,41,70,71]. The acid phosphatase activity is found in different cell types and has been associated with intracellular digestion, including the degradation of material internalized by cells [28,33,71,72]. The high activity of this enzyme, also named Lysosomal Acid Phosphatase (LAP), has been detected under certain circumstances, such as formation of tissues and organs including rat nervous tissue [73] and amphibian vitelline vesicle [74], as well as in the tissue remodelling, such as in rat endometrium [29] and periodontium [28], reinforcing the concept that the acid phosphatase plays an important role in the tissue degradation/remodelling. Moreover, the ACP-2 knockout promotes skeletal abnormalities during rat development [75], and mutations in ACP-2 gene are associated with cerebellar malformations, suggesting a critical role of ACP-2 in tissue development [33]. Here, the positive reaction to acid phosphatase was present in the fibroblasts and macrophages, mainly in the 13- and 16-day-old rats, i.e., at mucosal penetration stage of the tooth eruption. Usually, these cells were surrounded by extracellular matrix exhibiting a granular and flocculent material, indicating the occurrence of degradation of its components. The presence of material inside macrophage and fibroblast vacuoles exhibiting acid phosphatase activity indicates that these cells may be digesting the remnants of extracellular matrix. Therefore, the ECM components are degraded by different MMPs, including MMP-1, and the remnants are engulfed and digested by macrophages and fibroblasts. Once inside a vacuole, the remnants are degraded by lysosomal enzymes such as acid phosphatase (Figure 10). Moreover, we cannot exclude the possibility that acid phosphatase reactivity inside large vacuoles may also be involved in the intracellular digestion of apoptotic bodies, as described in other tissues and organs [76,77]. It is known that changes in the ECM affect cell survival and proliferation since the interactions between ECM components and cell surface molecules regulate cell behaviour [42,78]. ECM fragments derived from cleavage by MMPs induce apoptosis of mammary epithelial cells [79], indicating that changes in the ECM microenvironment may promote apoptosis. Here, an enhanced immunoexpression of MMP-1 and ACP-2 was detected in the eruptive pathway at the mucosal penetration phase (13- and 16-day-old rats), suggesting a coordinate interaction between theses enzymes since MMP-1 cleaves ECM substrates, and ACP-2 is responsible for intracellular digestion (Figure 10). In fact, the number of apoptotic cells in the lamina propria increases significantly at the mucosal penetration phase of eruption when macrophages and fibroblasts engulfing apoptotic bodies were found [10], and acid phosphatase activity was observed inside large vacuoles containing digesting remnants material. Therefore, the establishment of the eruptive pathway for the passage of erupting teeth involves a coordinated cascade of cellular and molecular events, culminating in a rapid and programmed degradation of cellular and ECM components of the lamina propria.

In conclusion, the collagen content of the lamina propria reduces significantly during tooth eruption due to degradation of the extracellular matrix components by the MMP-1. The enhanced acid phosphatase activity in the fibroblasts and macrophages of the lamina propria during the mucosal penetration stage points to an important role of this enzyme in the intracellular digestion of extracellular remnants, being involved in the establishment of the eruptive pathway. Although the present study was performed in rodent model, it is well stated that the tooth eruption in rat molars is similar to human, including the stages of eruption. Thus, despite the limitations of extrapolating the results obtained in rodents to humans, our findings indicate that any disturb in MMP-1 and/or ACP-2 expression may delay or impair tooth eruption. Further studies, including the evaluation of the control and participation of MMPs and ACPs during tooth eruption are necessary for the better understanding of the causes of delayed tooth eruption, mainly when this disturb is not of physical origin.
